# Dr. Irving O. Nellis

**Published:** 1916-05

**Authors:** 


**Dr. Irving O. Nellis,** Vermont 1882, died at his home in Utica, March 8, aged 59. He had formerly resided in Herkimer and was at various times coroner, health officer, president of the Herkimer Board of Health and deputy sheriff of the county.

